# Editorial: Advancements of deep learning in medical imaging for neurodegenerative diseases

**DOI:** 10.3389/fnins.2024.1361055

**Published:** 2024-01-19

**Authors:** Loveleen Gaur, Patrick Siarry, Ajith Abraham, Oscar Castillo

**Affiliations:** ^1^Department of Computer Science, Taylor's University, Subang Jaya, Malaysia; ^2^Department of Computer Science, Universite Paris 12, Creteil, France; ^3^Department of Computer Science, Machine Intelligence Research Labs, Auburn, AL, United States; ^4^Instituto Tecnológico de Tijuana, Tijuana, Mexico

**Keywords:** artificial intelligence, neurodegenerative diseases, deep learning, Alzheimer's disease, Parkinson's disease

## Introduction

The integration of technology and healthcare, particularly through artificial intelligence and deep learning, is revolutionizing precision medicine. Advances in medical imaging using deep learning have proven effective in diagnosing and managing neurodegenerative diseases like Alzheimer's, Parkinson's, and Multiple Sclerosis (Noor et al., 2019; Myszczynska et al., 2020; Gaur et al., 2023; Ghose et al., 2023; Xu et al., 2023). Deep learning excels in detecting subtle changes in brain structure and function, providing early detection, and significantly influencing patient prognosis and treatment efficacy (Tarnanas et al., 2022; Jyotismita and Marcin, 2023; Modat et al., 2023). This transformative synergy enhances our understanding of disease progression and marks a crucial advancement in neurodegenerative disease diagnosis and management (Breijyeh and Karaman, 2002; Buergel et al., 2022; Gaur et al., 2022a,b; Ghose et al., 2022). This editorial aims to illuminate the remarkable strides made in the field of medical imaging, specifically the contributions of deep learning, in unraveling the complexities of neurodegenerative diseases.

The article titled “*A diagnosis model for brain atrophy using deep learning and MRI of type 2 diabetes mellitus*” authored by Syed and M. A. aims to develop a sophisticated diagnostic model for identifying and characterizing brain atrophy in Type 2 Diabetes Mellitus (T2DM). Leveraging a dataset of 235 MRI images affected by T2DM-induced brain atrophy, the researchers meticulously divided the dataset for training and testing. Using advanced techniques like the TRAU-Net model for segmentation and Multinomial Logistic Regression with Attention Swin Transformer (MLAST) for classification, the model demonstrated exceptional performance metrics, surpassing existing counterparts. The study concludes optimistically, highlighting the potential deployment of this automated diagnostic model in healthcare for nuanced assessments of T2DM-related brain atrophy, marking significant progress in clinical diagnostics.

“*Learning from pseudo-labels: deep networks improve consistency in longitudinal brain volume estimation*” by Zhan et al. addresses challenges in measuring brain atrophy, a crucial biomarker in neurodegenerative diseases. Inconsistent imaging acquisitions hinder precise assessments, prompting the development and validation of DeepBVC, a resilient deep learning model. Utilizing voxel-wise pseudo-atrophy labels generated by SIENA, the study involves 195 pairs of longitudinal 3D T1 scans from multiple sclerosis (MS) patients. DeepBVC, a 3D U-Net, excels in overcoming scan variations, outperforming SIENA in median percent brain volume change. The study underscores DeepBVC's reproducibility and potential in research and clinical settings, offering enhanced measurement robustness and automation for monitoring disease progression and treatment evaluation.

“*A novel framework of MOPSO-GDM in recognition of Alzheimer's EEG-based functional network*” by Wang et al. study introduces an innovative EEG-based framework for Alzheimer's recognition, employing deep learning methods. The methodology incorporates network interactions in various frequency bands, reconstructing multiplex networks with the PSI method and extracting fourteen topology features. To address feature inefficiency and manual selection challenges, the proposed MOPSO-GDM algorithm combines MOPSO with GDM, optimizing classification accuracy and distance measures. Results demonstrate a remarkable 5.31% classification accuracy with an 8-feature vector, showcasing the framework's adaptability and potential for efficient disease recognition, contributing to a comprehensive understanding of brain function in neurological disorders.

“*Identifying key multi-modal predictors of incipient dementia in Parkinson's disease: a machine learning analysis and Tree SHAP interpretation*” by McFall et al.. In a longitudinal study of Parkinson's disease (PD) patients without dementia, McFall et al. utilized advanced machine learning to identify predictors of subsequent dementia development. The research, involving 48 well-characterized PD patients and 38 predictors, employed Random Forest models, achieving an AUC of 0.84 in discriminating PD patients who developed dementia. Tree SHapley Additive exPlanation values revealed ten leading predictors from various domains, emphasizing the multifaceted nature of dementia risk in PD patients. This data-driven approach successfully identified and interpreted early predictors, enhancing understanding in diverse risk domains.

“*Multi-modal feature selection with anchor graph for Alzheimer's disease*” by Li et al. addresses the need for improved diagnostic methods in Alzheimer's disease research. Current approaches often overlook interrelationships between data sources, modality significance, and local data structures. The innovative algorithm introduced employs an anchor graph, enhancing multi-modal feature selection by incorporating modal weights based on least square loss and l2,1–norm. The methodology, validated using the ADNI dataset, demonstrates potential in advancing Alzheimer's disease diagnosis through improved consideration of inter-modal relationships and local data structures.

These papers highlight the crucial role of advanced technologies, particularly machine learning, deep learning, and artificial intelligence, in enhancing our understanding and early detection of neurodegenerative disorders like Alzheimer's and Parkinson's disease. Integrating these technologies improves diagnostic accuracy, providing profound insights into the intricate nature of these conditions. Overall, these studies advance diagnostic tools and broaden our understanding of neurodegenerative diseases, highlighting interdisciplinary potential to revolutionize neurological research and clinical practice.

## Author contributions

LG: Conceptualization, Writing—original draft, Writing—review & editing. PS: Writing—review & editing. AA: Writing—review & editing. OC: Writing—review & editing.
